# Photoinduced Desorption Dynamics of CO from Pd(111): A Neural Network Approach

**DOI:** 10.1021/acs.jctc.1c00347

**Published:** 2021-07-19

**Authors:** Alfredo Serrano Jiménez, Alberto P. Sánchez Muzas, Yaolong Zhang, Juraj Ovčar, Bin Jiang, Ivor Lončarić, J. Iñaki Juaristi, Maite Alducin

**Affiliations:** † Centro de Física de Materiales CFM/MPC (CSIC-UPV/EHU), Paseo Manuel de Lardizabal 5, 20018 Donostia-San Sebastián, Spain; ‡ Hefei National Laboratory for Physical Science at the Microscale, Key Laboratory of Surface and Interface Chemistry and Energy Catalysis of Anhui Higher Education Institutes, Department of Chemical Physics, University of Science and Technology of China, Hefei, Anhui 230026, China; § Ruđer Bošković Institute, Bijenička 54, HR-10000 Zagreb, Croatia; ∥ Donostia International Physics Center (DIPC), Paseo Manuel de Lardizabal 4, 20018 Donostia-San Sebastián, Spain; ⊥ Departamento de Polímeros y Materiales Avanzados: Física, Química y Tecnología, Facultad de Químicas (UPV/EHU), Apartado 1072, 20080 Donostia-San Sebastián, Spain

## Abstract

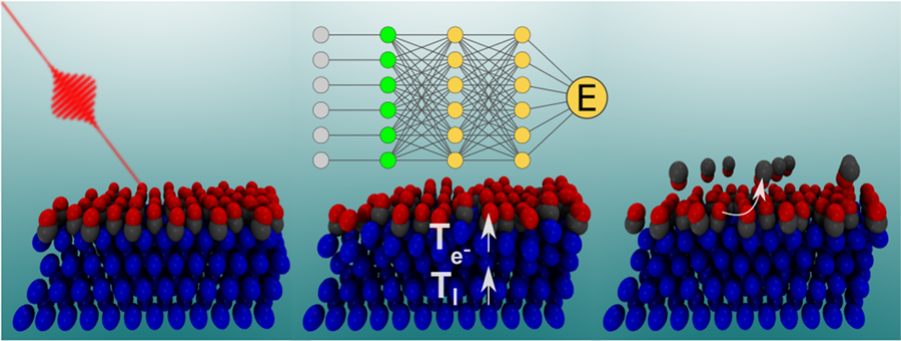

Modeling the ultrafast photoinduced dynamics and reactivity of adsorbates on metals requires including the effect of the laser-excited electrons and, in many cases, also the effect of the highly excited surface lattice. Although the recent ab initio molecular dynamics with electronic friction and thermostats, (*T*_e_,*T*_l_)-AIMDEF [AlducinM.;Phys. Rev. Lett.2019, 123, 246802]3192286010.1103/PhysRevLett.123.246802, enables such complex modeling, its computational cost may limit its applicability. Here, we use the new embedded atom neural network (EANN) method [ZhangY.;J. Phys. Chem. Lett.2019, 10, 49623139715710.1021/acs.jpclett.9b02037] to develop an accurate and extremely complex potential energy surface (PES) that allows us a detailed and reliable description of the photoinduced desorption of CO from the Pd(111) surface with a coverage of 0.75 monolayer. Molecular dynamics simulations performed on this EANN-PES reproduce the (*T*_e_,*T*_l_)-AIMDEF results with a remarkable level of accuracy. This demonstrates the outstanding performance of the obtained EANN-PES that is able to reproduce available density functional theory (DFT) data for an extensive range of surface temperatures (90–1000 K); a large number of degrees of freedom, those corresponding to six CO adsorbates and 24 moving surface atoms; and the varying CO coverage caused by the abundant desorption events.

## 1 Introduction

The use of intense (∼1 mJ/cm^2^) femtosecond (fs) laser pulses in the ultraviolet, visible, and near-infrared regime has been shown to be a very efficient way to promote reactions at adsorbate-covered metal surfaces.^1−3^ At these wavelengths, a large fraction of the light is absorbed by the metal giving rise to electronic excitations. Subsequently, energy transfer to the lattice atoms takes place via electron–phonon coupling. As a result, the adsorbates encounter a combined electronic and phononic excited system from which they can gain energy and experience different reactions, diffusion, and even desorption from the surface.^4−9^ Interestingly, this kind of excitation mechanism can increase significantly the cross section of reactions with respect to what is observed under ordinary thermal excitation conditions and even open new reaction channels. Two pulse correlation experiments are customarily employed to obtain the time scale of the energy transfer between the adsorbate and the substrate. In this way, in principle, whether a specific reaction is mainly governed by the excited electrons or phonons can be disentangled experimentally. However, in several cases, this information is not unequivocally obtained from the experiments and theoretical modeling is necessary.

From a theoretical aspect, the modeling of these experiments requires performing molecular dynamics simulations in an excited environment.^10−17^ First, the excitation generated by the laser pulse in the substrate is described in terms of time-dependent electronic (*T*_e_) and phononic (*T*_l_) temperatures that are obtained using the two-temperature model (2TM).^18^ Subsequently, the motion of the adsorbates is determined by solving Langevin equations of motion in the ground-state potential energy surface (PES). In this way, the coupling of the adsorbates to the electronic system is modeled in terms of electronic friction forces and associated stochastic forces that depend on *T*_e_. A nonempirical and accurate potential energy surface is typically obtained by characterizing the adsorbate–metal surface interaction at the level of density functional theory (DFT). Until very recently, due to the large computational cost involved in the DFT calculations, potential energy surfaces of reduced dimensionality, only involving the degrees of freedom (DOFs) of the adsorbate, were used. As a result, the effect of the heated phonon system was either not included at all in the dynamics or in a rather approximate way using the generalized Langevin oscillator (GLO) model that does not account for independent surface atom movement.^13−17^ Moreover, a maximum of two atomic adsorbates (or a single diatomic molecule) were described with six-dimensional potential energy surfaces, which did not allow one to model interadsorbate energy exchange and study effects related to the local reduction of the coverage that is caused by sequential desorption events.

In this respect, only very recently have these limitations been overcome using ab initio molecular dynamics with electronic friction (AIMDEF).^19−27^ The model, hereafter denoted as (*T*_e_,*T*_l_)-AIMDEF,^27^ incorporates both the electronic and phononic excitation channels: the former by solving the Langevin equation for the adsorbates using *T*_e_ and the latter by coupling the surface atoms to a thermostat at a temperature *T*_l_. This methodological approach naturally includes all of the system’s degrees of freedom as required. Specifically, in principle, any number of surface atoms are allowed to move independently and multiple adsorbates can be treated. Using (*T*_e_,*T*_l_)-AIMDEF, the importance of including not only *T*_e_ but also *T*_l_ (particularly for those surfaces that may reach a high *T*_l_), as well as the interadsorbate interactions, has been demonstrated.^27^ The main shortcoming of the approach is that AIMDEF is extremely computationally demanding. This means that, in practice, a reduced number of the order of few hundreds of trajectories can be realistically computed for a given set of experimental conditions. This results in limited statistics. For the same reason, with the required time steps of the order of femtoseconds, the integration time is limited to around 2–4 ps.

In the last few years, the use of neural network (NN)-generated multidimensional PESs has become an accurate alternative to ab initio molecular dynamics (AIMD) to describe the dynamics of diverse gas–surface processes^28−39^ and also the dynamics at solid–liquid water interfaces.^40−43^ In particular, for these studies, the development of the atomistic neural network (AtNN) approach has constituted a major advancement.^44−46^ Within AtNN, the PES is constructed in terms of atomistic contributions, which allows for obtaining NN-PESs that are a function of all the atomic positions in systems of arbitrary size. Application of this methodology to gas–surface dynamics studies has allowed for constructing PESs not only for diatomic^31−33,35,39^ but also for polyatomic molecules^29,30,34,36,37^ interacting with surfaces. Moreover, since the NN-PES can also be made a function of the surface atom coordinates, the treatment of both the independent surface atom movement and the surface temperature effects has also been performed, which has allowed for accounting for energy exchange between the molecule and the surface along the dynamics.^31−33,35−39^

However, it must be emphasized that the requirements imposed on a NN-PES capable of describing femtosecond laser-induced reactions are extremely demanding as compared to those required in usual elementary gas–surface processes. Since it is necessary to model the movement of multiple adsorbates and surface atoms, the number of degrees of freedom to be accounted for is huge. In this respect, it is worth mentioning that, to our knowledge, all the NN-PESs generated up to now for gas–surface studies are restricted to a single gas molecule. Moreover, the PES must be able to describe accurately the very distinct and changing adsorbate coverages that exist during the photoinduced dynamics. This means that it is necessary to ensure a precise description of adsorbate–substrate and interadsorbate interactions under very different conditions involving local changes of the configuration space of neighbor adsorbates and strong lattice distortions. In this respect, note that in these experiments, the lattice temperatures (*T*_l_) may vary rapidly in the range of 90–1000 K, which implies that the configuration space corresponding to the surface atoms is very large. Therefore, altogether the requirements for the atomistic NN-PES are unprecedentedly extreme and demanding for these kinds of processes. Here, we show that the recently developed embedded atom neural network (EANN) method,^47^ which has already been successfully applied to construct the PESs for diatomic and polyatomic molecules interacting with multiple metal facets,^48,49^ is indeed impressively accurate and flexible to account for all these necessities.

For this purpose, we study the femtosecond laser-induced desorption of CO from Pd(111).^9^ In particular, we concentrate on the CO saturation coverage of 0.75 monolayer (ML), in which the CO molecules adsorb in atop, face-centered cubic (fcc), and hexagonal close-packed (hcp) sites. (*T*_e_,*T*_l_)-AIMDEF results for this system were recently presented in ref (27). In the present work, we use the configurations encountered along these dynamics as the input data to generate our EANN-PES.

The work is organized as follows. In Section 2, the procedure used to construct EANN-PES is detailed and accuracy tests are presented. Next, Section 3 is devoted to the description of the theoretical framework used to perform molecular dynamics simulations of the laser-induced reactions at metal surfaces. Also, in this section, we present the method used to obtain the background electronic density at the position of the moving adsorbates at each time step of the dynamics, which is required to obtain the friction coefficients that describe the coupling of the adsorbates to the heated electronic system in the Langevin equation of motion. Subsequently, in Section 4, the molecular dynamics simulations performed in the precalculated 0.75 ML-CO/Pd(111) EANN-PES to model the laser-induced desorption of CO from the Pd(111) surface are presented. The results of the simulations are compared to the (*T*_e_,*T*_l_)-AIMDEF results of ref (27) showing, conclusively, the validity of our EANN-PES to perform molecular dynamics in these unprecedentedly exigent conditions. Finally, in Section 5, the main conclusions of the work are summarized.

## 2 EANN-PES Generation and Quality Check

The analytical representation of the adiabatic PES *E*({**r**_*k*_}) ruling the desorption of CO from Pd(111) with 0.75 ML coverage is calculated with the recently developed embedded atom neural network (EANN) method.^47^ Similar to AtNN by Behler and Parrinello,^44^ the total energy of an *N* atom system is expressed as the sum of the energy of each atom that conforms it, *E*_*i*_({**r**_*k*_}). In the EANN framework, *E*_*i*_({**r**_*k*_}) is described in terms of the electronic embedding density, i.e.
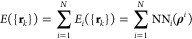
1where NN_*i*_ is the species-dependent atomic neural network of the *i*th atom in the system that depends on the embedding density vector **ρ**^*i*^ whose components represent the local electron density provided by the surrounding near atoms. Specifically, the set of local density components defining **ρ**^*i*^ are given by
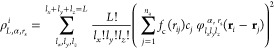
2where
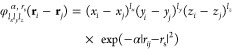
3are Gaussian-type orbitals (GTOs) centered at each of the *n*_a_ atoms *j* that are located within a radius *r*_c_ from the embedded atom *i*. In these equations, **r**_*i*_ = (*x*_*i*_, *y*_*i*_, *z*_*i*_) and **r**_*j*_ = (*x*_*j*_, *y*_*j*_, *z*_*j*_) are the Cartesian position vectors of atoms *i* and *j*, respectively, with *r*_*ij*_ = |**r**_*i*_ – **r**_*j*_|; α and *r*_s_ determine the width and the center of the Gaussian-like term in eq 3, respectively (and thus control the shape of the radial distribution related to each GTO), while *l*_*x*_, *l*_*y*_, and *l*_*z*_ are the values of the orbital angular momentum in each coordinate, whose sum equals the total angular momentum *L*, i.e., *L* = *l*_*x*_ + *l*_*y*_ + *l*_*z*_. In eq 2, *f*_c_(*r*_*ij*_) is the commonly used cosine type cutoff function that makes the interaction to smoothly decay to zero as *r*_*ij*_ approaches the cutoff radius *r*_c_,^44^ and *c*_*j*_ is the element- and orbital-dependent weight^50^ adjusted during the NN_*i*_ training. Each *c*_*j*_ can be regarded as the expansion coefficient of the orbital φ_*l*_*x*_*l*_*y*_*l*_*z*__^α,*r*_s_^ of atom *j*.

**Figure 1:**
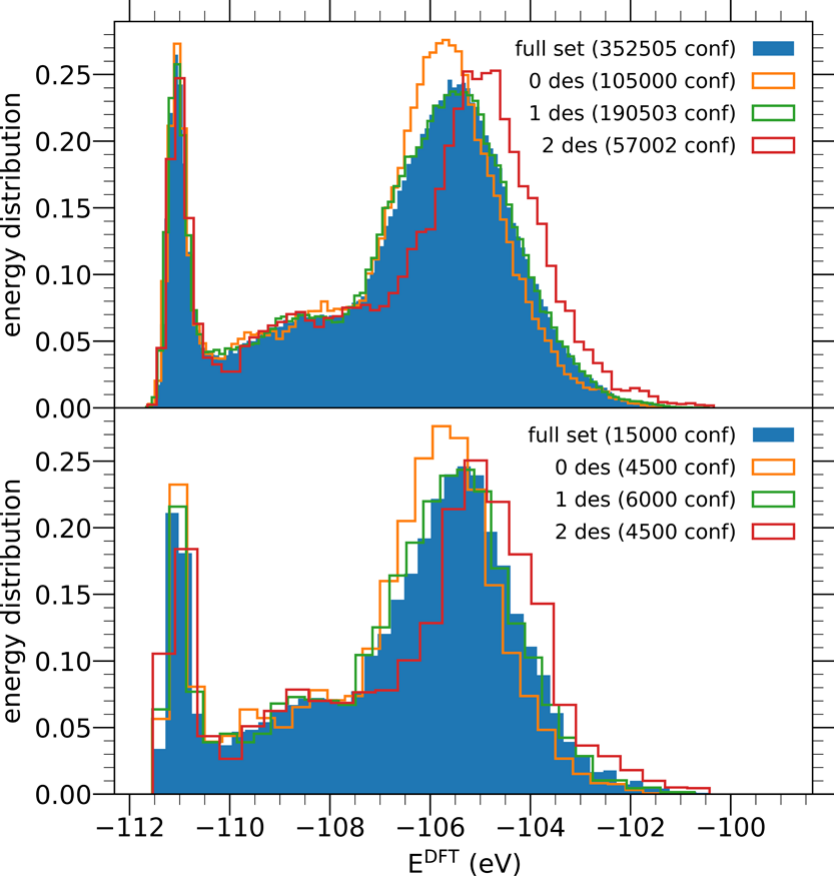
Normalized DFT potential energy distributions extracted from (*T*_e_,*T*_l_)-AIMDEF simulations^27^ (filled blue bars). The sets of energies corresponding to trajectories with zero, one, and two CO desorption events are plotted in yellow, green, and red empty histogram bars, respectively. Top: distributions for the whole AIMDEF data set (352 505 configurations). Bottom: distributions for the 15 000 configurations used for the first EANN-PES training.

The atomic configurations in the reference data set are extracted from the (*T*_e_,*T*_l_)-AIMDEF simulations of the photoinduced desorption of CO from the Pd(111) surface with 0.75 ML coverage performed in ref (27). These DFT calculations were performed with the Vienna Ab initio Simulation Package (VASP)^51,52^ (version 5.4), using the van der Waals density functional (vdW-DF) exchange–correlation functional proposed by Dion et al.,^53^ and the AIMDEF module^20−26^ that was extended to include excited electrons and excited phonons effects through time-dependent electronic and lattice temperatures (see Section 3).^27^ A total of 100 trajectories were calculated in a four-layer (4 × 2) supercell that contained six CO adsorbates equally distributed among atop, hcp, and fcc sites and 32 Pd atoms describing the Pd(111) surface. Note in passing that the use of such a large cell (twice the unit cell for 0.75 ML) was aimed to account for out-of-phase movements of the coadsorbed CO molecules, providing a more realistic description of the interadsorbate interactions. Each trajectory was integrated up to 3.5–4 ps using a time step of 1 fs. Altogether, the whole data set consists of 352 505 configurations, each defined by the position vectors of the 44 atoms in cell {**r**_*i*_}, for which the corresponding DFT potential energies *E*^DFT^ and DFT forces on each atom *F*_*i*_^DFT^ are well characterized. The training process can be greatly simplified if, instead of using such a huge amount of data, we select a smaller subset that correctly represents the relevant configurational space of the system. There are different properties that can guide this selection, e.g., distance of the gas species to the surface,^31^ forces,^37^ etc. In our case, the photoinduced desorption dynamics is characterized by trajectories that yield none, one, or two desorbing CO, each conceivably providing information on the interaction at variable coverages. Thus, an initial subset is constructed with 4500, 6000, and 4500 configurations that are randomly selected from the set of trajectories with zero, one, and two desorption events, respectively. The ulterior analysis of the covered potential energy range served us to validate that the configurational space probed in the AIMDEF simulations is in principle well sampled. Figure 1 shows that the *E*^DFT^ distributions of the whole and of each of the three types of desorbing trajectories (top panel) are accurately reproduced by the subset of 15 000 trajectories (bottom panel). It is worth mentioning that the values *E*^DFT^ in Figure 1 are the output of the VASP program. As shown in this figure, the relevant potential energy variation range in the dynamical configurational space covers around 12 eV, which corresponds to around 0.3 eV per moving atom (see below).

The analytical EANN-PES for the 0.75 ML-CO/Pd(111) system uses 60 density descriptors for each atomic species that correspond to take *L* = 0–3 combined with 15 Gaussian functions with α = 0.93 Å^–2^ and *r*_s_ varying within the interval [0,*r*_c_] in increments Δ*r*_s_ = 0.46 Å for our chosen value *r*_c_ = 6.5 Å. Different architectures were tried, but an optimal balance between small errors in energy and the required computation time is achieved using two hidden layers with 60 neurons each for every atomic NN_*i*_. The EANN code takes advantage of the usual random separation of the reference data set into training and validation subsets to produce different NNs during the same run. In our case, five EANN-PESs have been trained using 90 and 10% of the configurations as training and validation subsets, respectively. An efficient extreme machine learning Levenberg–Marquardt algorithm is used in the optimization of the fitting parameters (*c*_*j*_).^54^ The cost function used to evaluate at each iteration the quality of an EANN-PES involves the energies and atomic forces. As discussed in ref (47), convergence is very efficient in the EANN method. For our settings, the required accuracy is achieved in the five training PESs in less than 50 iterations, being the root-mean-square-errors (RMSEs) in the energy per moving atom of 0.43–0.58 and 0.90–1.05 meV for the training and validation sets, respectively.

**Figure 2:**
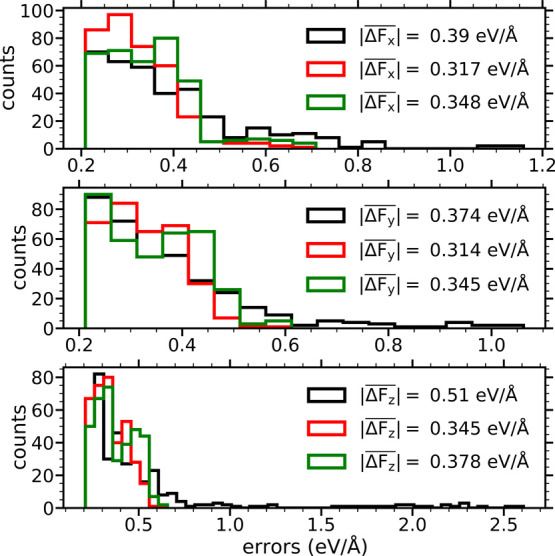
Error histograms for each Cartesian component of the atomic forces, |Δ*F*_β_| = |*F*_β_^DFT^ – *F*_β_^EANN^| (where β = *x*, *y*, *z*), calculated using only the 10 maximum error values for each of the 36 moving atoms of the 0.75 ML-CO/Pd(111) system. Black (red) histogram bars correspond to errors for the predict set in the best EANN-PES of the first (final) training. Green histogram bars show the maximum errors for the whole AIMDEF data set of 352 505 configurations as obtained with the final EANN-PES. The mean values  of each histogram are also provided.

Since the photoinduced desorption dynamics of interest implies that the system is exposed to extreme conditions characterized by high surface temperatures (1000 K) and highly excited adsorbates, we consider it important to evaluate the accuracy of the PES in predicting not only the energy but also, especially, the atomic forces. A new data set formed by 87 382 configurations randomly taken from the AIMDEF data set not used in the fitting process and representative of the three types of desorbing trajectories is used for this purpose (predict data set). The results show that the obtained PESs are excellent with the RMSE in energy of only 0.86–0.95 meV per moving atom and the RMSE in the Cartesian components of the forces of 0.05–0.06 eV/Å. Nevertheless, we observe that the maximum errors in the forces are in some cases large (between 1.1 and 5.9 eV/Å depending on the coordinate and trained EANN-PES considered). Thus, new configurations are added to the initial set of 15 000 structures to improve the quality of our EANN-PES. The criterion to select these new configurations is as follows. First, among the five trained EANN-PESs, we select the one with the smallest maximum absolute errors in the atomic forces, i.e., |Δ*F*_β_| = |*F*_β_^EANN^ – *F*_β_^DFT^|, where β = *x*, *y*, *z* refers to the force component. From this analysis, we select the 10 largest errors for each of the 36 moving atoms (i.e., 12 atoms forming the six CO adsorbates and 24 Pd atoms that correspond to the moving three of the four layers describing the surface). Figure 2 shows the distribution of these maximum errors for each of the force components as obtained with the EANN-PES with the smallest errors in the forces (black histogram bars). The corresponding configurations (883 because errors in different force coordinates can occur for the same configuration) are added to the original 15 000 data input to develop five new EANN-PESs, using the same NN settings (i.e., basis set and architecture).

**Figure 3:**
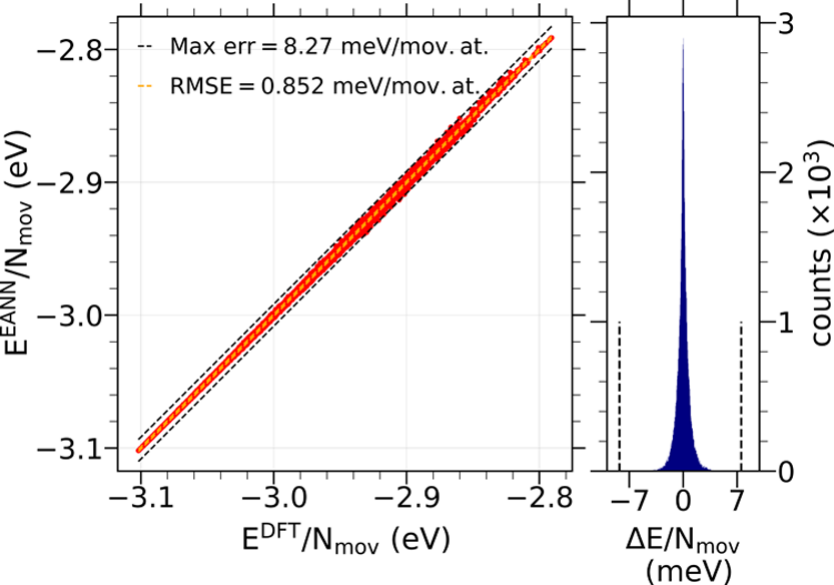
Left: comparison of the potential energies per moving atom computed with the (final) EANN-PES for a set of 87 382 configurations not used in the training and the corresponding DFT values. Right: histogram of the errors in energy per moving atom Δ*E*/*N*_mov_ = (*E*^DFT^ – *E*^EANN^)/*N*_mov_ for the same set of predict configurations.

The quality of the five newly developed EANN-PESs is impressively good. Since the accuracy in the forces is also crucial for reproducing the system dynamics and thus its physical behavior properly, we select the EANN-PES with the smallest errors in the atomic forces. In this PES, the RMSE in the energies per moving atom is 0.41 and 0.87 meV for the training and validation sets, respectively. Its accuracy is further confirmed by the energy results obtained with the predict data set. As shown in Figure 3 (left panel), the maximum error per moving atom is 8.27 meV but the small RMSE of 0.85 meV marks the minor error introduced in most of the configurations. The error distribution plotted in Figure 3 (right panel) clearly shows that this is the case.

**Figure 4:**
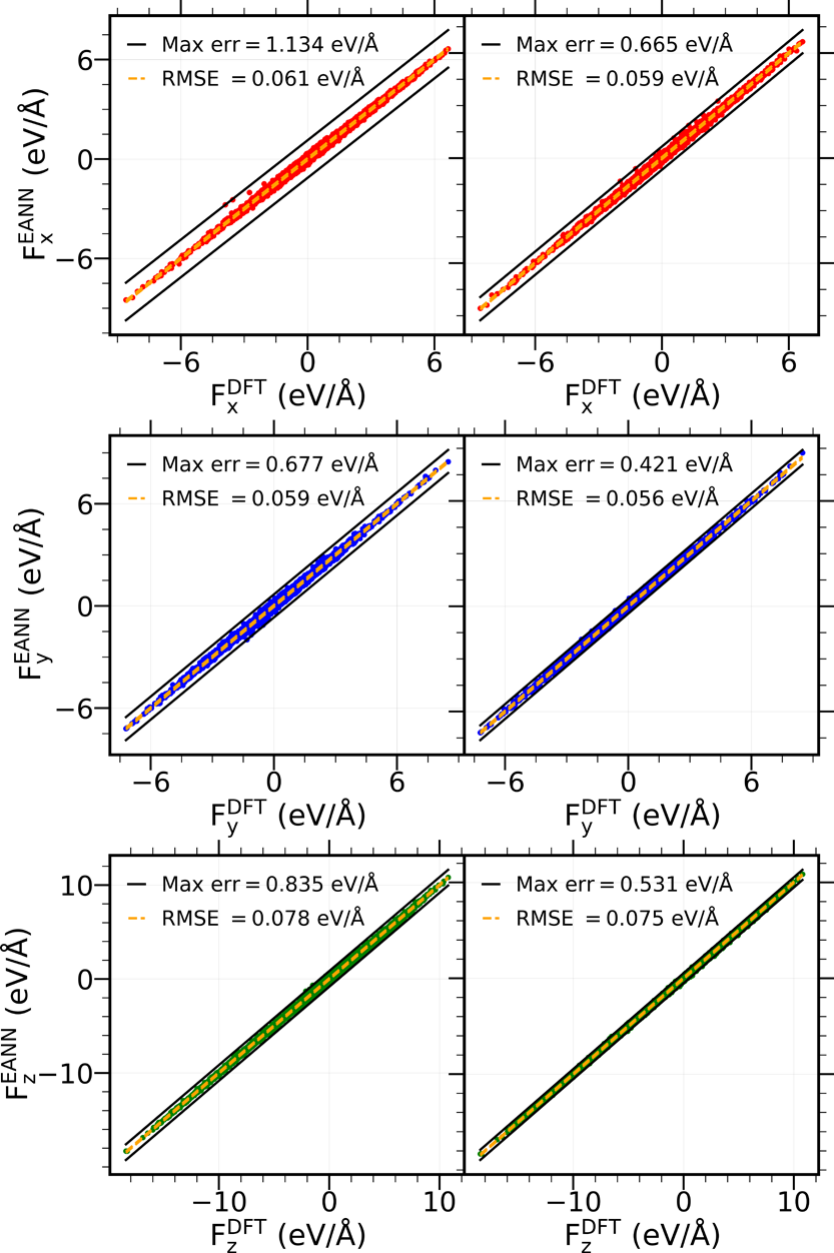
Plots of *F*_β_^EANN^ versus *F*_β_^DFT^ for a fcc C atom of the 0.75 ML-CO/Pd(111) system^27^ showing a comparison for each Cartesian component β = *x* (red), *y* (blue), and *z* (green). The left panels correspond to forces calculated with the first trained EANN-PES and the right panels to those calculated with the final EANN-PES. Maximum error and RMSE of the forces are written in each plot.

**Figure 5:**
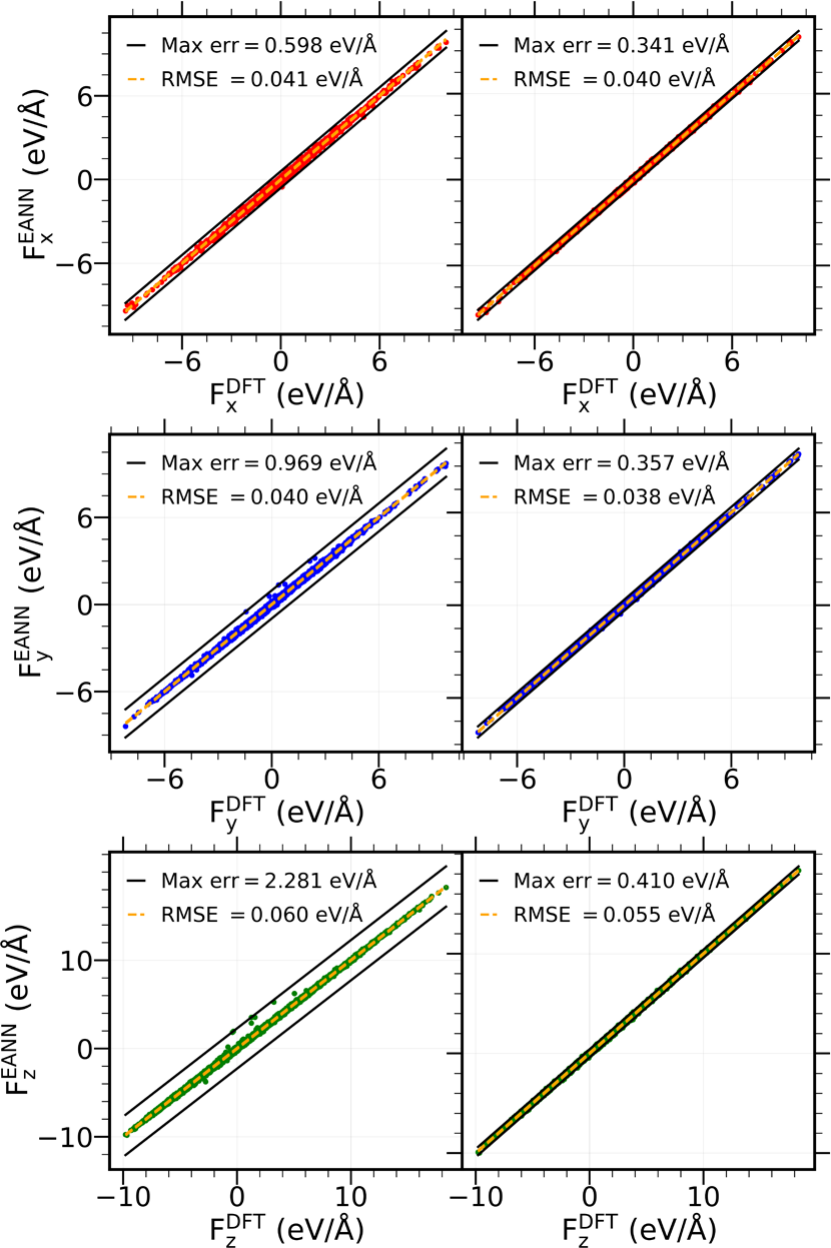
Same as Figure 4 for a hcp O atom.

Regarding its accuracy in predicting the atomic forces, the RMSE is not greatly improved in the retraining process and it is still around 0.05 eV/Å, whereas the mean absolute errors are around 0.04 eV/Å. However, a comparison of the 10 maximum absolute errors for each atomic force component |Δ*F*_β_| obtained with our final EANN-PES (red bars in Figure 2) with the ones of the first training process (black bars) shows a remarkable reduction in the errors. The reduction in the maximum errors is as follows: |Δ*F*_*x*_| ≈ 1.13 → 0.66 eV/Å, |Δ*F*_*y*_| ≈ 1.04 → 0.58 eV/Å, and |Δ*F*_*z*_| ≈ 2.56 → 0.62 eV/Å. This improvement is also noticeable when comparing the mean absolute error (taken over the 10 maximum errors for each of the 36 moving atoms) of *F*_*z*_, plotted in black and red histograms, that decreases from . The averaged errors were already small for the two other components of the forces and in this improved EANN-PES are just slightly reduced,  and . As an additional stringent test, we also show the maximum error predictions of the final EANN-PES on the 352 505 configurations forming the full AIMDEF data set (green bars of Figure 2). In this case, we obtain |Δ*F*_*x*_| = 0.68 eV/Å, |Δ*F*_*y*_| = 0.58 eV/Å, and |Δ*F*_*z*_| = 0.63 eV/Å so that the maximum absolute-valued errors show none to a very little increase with respect to the predictions of the red histogram. The corresponding mean absolute error values are slightly larger: , and . The results from all these validation tests imply that we should also observe good convergence regarding any atom of the CO/Pd(111) cell. Figures 4 and 5 show two examples of these good agreements between *F*_β_^EANN^ and *F*_β_^DFT^, β = *x*, *y*, *z*, for two representative atoms of the system: one C atom from a CO at a fcc site and one O from a hcp site-placed CO. As a result, after the full static analysis, we are confident about this final EANN-PES’s robustness, which we further determine by trying to reproduce the dynamics of the CO/Pd(111) system as described in ref (27).

## 3 Photoinduced Molecular Dynamics on Surfaces

**Figure 6:**
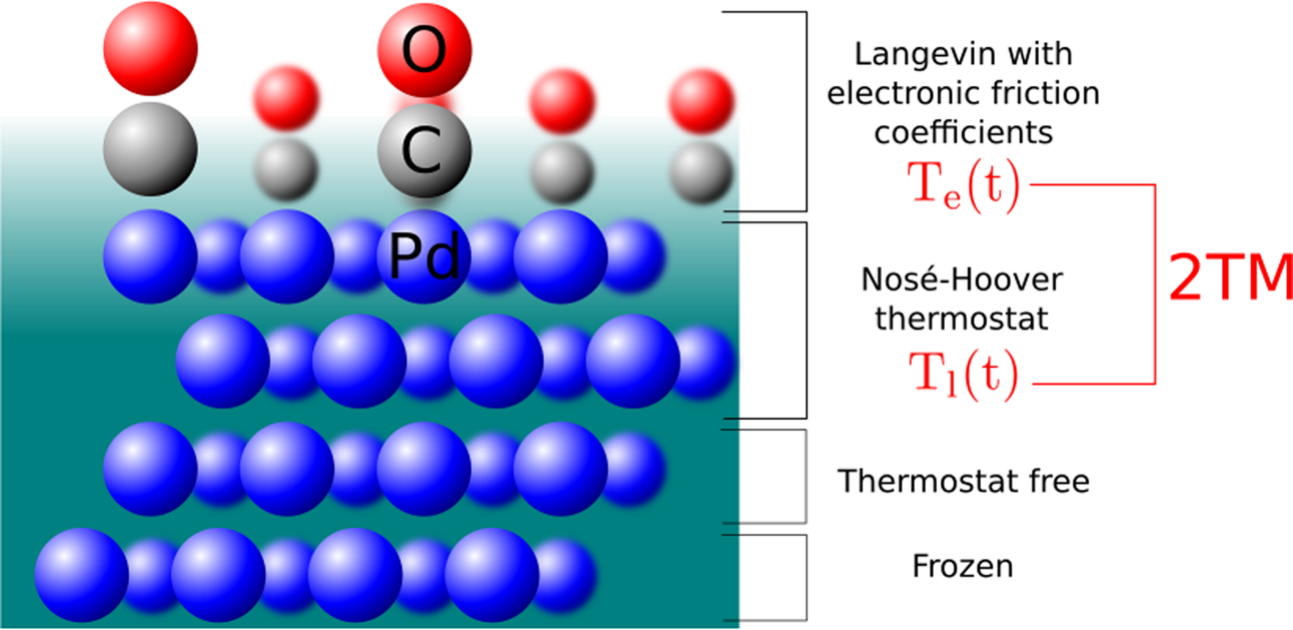
Scheme of the dynamics model used to simulate laser-induced desorption of CO from Pd(111) with 0.75 ML coverage.

The desorption of CO from the Pd(111) surface induced by femtosecond laser pulses is simulated with molecular dynamics with electronic friction and thermostat [(*T*_e_,*T*_l_)-MDEF] calculations performed in our developed EANN-PES. To do so, we have modified our implementation of the (*T*_e_,*T*_l_)-AIMDEF methodology^27^ to compute trajectories in which forces and electronic friction coefficients are evaluated at each visited configuration from an arbitrary PES and an arbitrary electronic density generator function (DGF), respectively. In Figure 6, we show a concise scheme of the (*T*_e_,*T*_l_)-(AI)MDEF model. Macroscopically, a laser pulse heats the electrons of the metal surface, which subsequently excite surface phonons. This response is modeled with the same 2TM as in the original (*T*_e_,*T*_l_)-AIMDEF calculations. The macroscopic response of the system is thus followed microscopically by CO and Pd degrees of freedom (DOFs) with different equations of motion.

CO DOFs are subjected to the following Langevin equations of motion
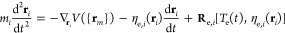
4where *m*_*i*_, **r**_*i*_, and η_e,*i*_ are the mass, position vector, and the electronic friction coefficient of the *i*th atom conforming the set of adsorbates, respectively. The first term on the right-hand side of the equation represents the adiabatic force that depends on the position of all (adsorbates and surface) atoms. In the (*T*_e_,*T*_l_)-AIMDEF simulations of ref (27), this force was calculated on the fly using the Hellmann–Feynman theorem. In the (*T*_e_,*T*_l_)-MDEF calculations that we present here, this force is calculated using the precalculated EANN-PES. The third term **R**_e,*i*_ is the random fluctuating force that mimics the effect of the hot metal electrons on the adsorbates, and it is responsible for the excitation of the latter by the excited electronic system. This force is related to the electronic friction force (second term) through the fluctuation–dissipation theorem. Specifically, **R**_e,*i*_ is modeled by Gaussian white noise with variance
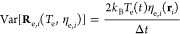
5where *k*_B_ and Δ*t* are the Boltzmann constant and the time-integration step, respectively.

The equations of motion followed by the Pd surface atoms incorporate two kinds of force terms. The first term consists of the adiabatic force that, as in the case of adsorbates, depends on the position of all atoms. Again, in the (*T*_e_,*T*_l_)-AIMDEF simulations of ref (27), this force is calculated on the fly using the Hellmann–Feynman theorem, whereas in the (*T*_e_,*T*_l_)-MDEF calculations, it is computed using our EANN-PES. The second term accounts for the heating of the surface layers due to the electronic excitation generated by the laser pulse. As done in ref (27), this effect is simulated by coupling the atoms of the two topmost surface layers to a thermal bath described by a Nosé–Hoover thermostat,^55,56^ in which the temperature is the time-dependent temperature *T*_l_(*t*) obtained from the 2TM. Thus, the equations of motion of these surface atoms *j* with mass *m*_*j*_ and position vector **r**_*j*_ are as follows
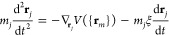
6
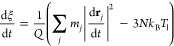
7where *N* is the number of atoms of the first two layers in our simulation cell, *Q* is a parameter with dimensions of energy × time^2^ that acts as the mass of the dynamical variable *s*, and ξ = *Q*^–1^*sp*_s_ is the thermodynamic friction coefficient.^57^ Finally, the third layer is used as a transition region between the hot surface and the inner (not heated) bulk. Therefore, the movement of each atom *k* in the third surface layer is described by the classical Newton equations of motion and the adiabatic approximation
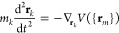
8where *m*_*k*_ and **r**_*k*_ are its corresponding mass and position vector, respectively. The fourth-layer atoms are kept frozen in our simulations.

Regarding the coupling of the adsorbates to the electronic system via the second and third terms in the right-hand side of eq 4, the electronic friction coefficient for each atom in each adsorbate η_e,*i*_ is calculated within the original local density friction approximation (LDFA)^19,58^ that in the case of molecular adsorbates treats the molecule as formed by independent atoms (independent atom approximation, IAA). This means that η_e,*i*_ depends on the value of the bare surface electronic density at the position of the atom *i* forming the adsorbate, i.e., *n*_sur_(**r**_*i*_). In the (*T*_e_,*T*_l_)-AIMDEF simulations,^27^ this is achieved using the Hirshfeld partitioning scheme^59^ to subtract the contribution of the adsorbates from the self-consistent electronic density that is calculated at each time step.^22,23^ In the case of (*T*_e_,*T*_l_)-MDEF, apart from an EANN-PES, the friction coefficients must also be obtained from precalculated results. Moreover, to use an EANN-PES efficiently, it is clear that the friction coefficients have to be calculated at least as fast as the potential. One could use a similar NN scheme to interpolate the density or the electronic friction coefficients based on the AIMDEF data set.^60^ Actually, even a more complicated object such as the electronic friction tensor can be interpolated by NNs.^61−63^ However, here we choose to employ a more simple approach that we show to be accurate and compared to a NN interpolation, faster and more stable.

We start by writing the Pd(111) electron density at the position of each atom of each CO adsorbate *n*(**r**_C,O_) as a sum of the electronic densities contributed by individual Pd atoms at this position
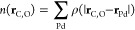
9We then assume that ρ(|**r**_C,O_ – **r**_Pd_|) can be described by two exponentially decaying functions
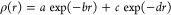
10Using AIMDEF data points of *n*(**r**_C,O_), one can fit the four parameters: *a*, *b*, *c*, and *d*. We did also try other functions such as Gaussian functions and their combination with the exponential function, but two decaying exponential functions gave the best results retaining the simplicity and a small number of parameters. Since it is easy to evaluate such a function, we decided to use all available (*T*_e_,*T*_l_)-AIMDEF surface electronic densities from which the corresponding friction coefficients were calculated. The fitting procedure results in the following parameters: *a* = 3.15975 au, *b* = 4.25214 Å^–1^, *c* = 0.29080 au, and *d* = 2.52252 Å^–1^ (au stands for atomic units).

Once the density *n*(**r**_C,O_) is known, the friction coefficients are calculated within the LDFA,^19,58^ as it was also done in the (*T*_e_,*T*_l_)-AIMDEF simulations. In particular, the C and O friction coefficients are individually fitted to the following analytical function
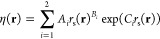
11where *r*_s_(**r**) = [3/(4π*n*(**r**))]^1/3^ is the mean electron radius. In eq 11, all the physical quantities are given in au, being the fitting parameters (*A*_1_ = 22.654, *B*_1_ = 2.004, *C*_1_ = −3.134, *A*_2_ = 2.497, *B*_2_ = −2.061, *C*_2_ = 0.0793) for C and (*A*_1_ = 1.36513, *B*_1_ = −1.8284, *C*_1_ = −0.0820301, *A*_2_ = 50.342, *B*_2_ = 0.490785, *C*_2_ = −2.70429) for O.

**Figure 7:**
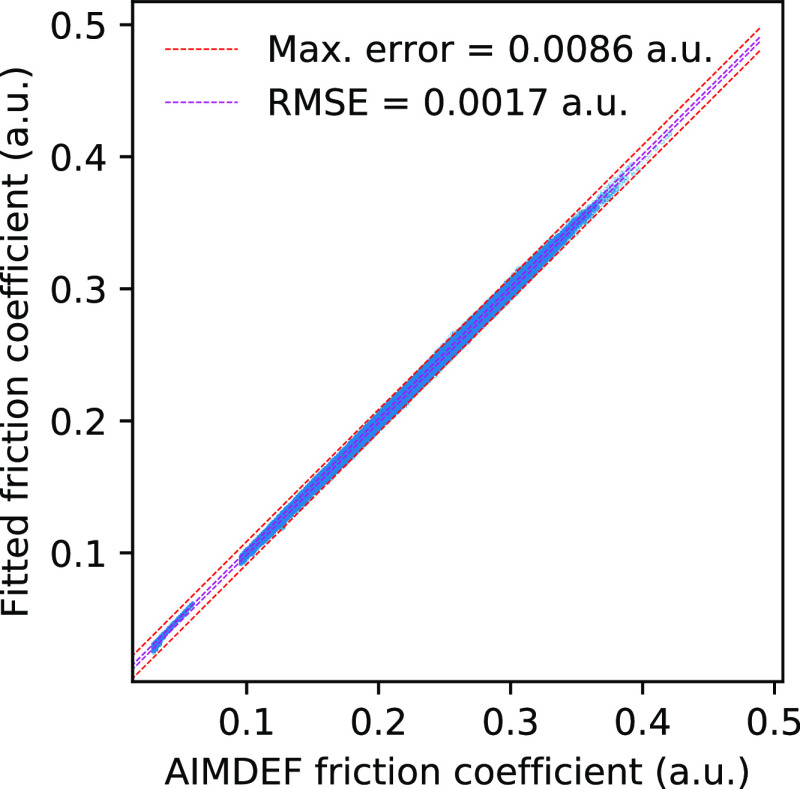
Comparison of the LDFA friction coefficients obtained from the fitted electronic density *n*(**r**_C,O_) (eqs 9 and 10) to all the available (*T*_e_,*T*_l_)-AIMDEF friction coefficient data. Friction coefficients given in atomic units (au).

In Figure 7, we compare the LDFA friction coefficients obtained with the fitted electron density *n*(**r**_C,O_) to their corresponding (*T*_e_,*T*_l_)-AIMDEF friction coefficients. It can be seen that both the maximum error of 0.0086 au and RMSE of 0.0017 au are small, especially compared to the errors associated with different calculations of embedding densities.^23^ In addition to accuracy, the advantage of this approach is that it is extremely fast in evaluating friction coefficients compared to the potential evaluation. Lastly, by the construction of two exponentially decaying functions, it is also ensured that there is no problem of overfitting or large errors in the extrapolation range that could occur with approaches such as NN. All this guarantees that eq 9 together with eq 10 constitutes an adequate DGF to be used in the (*T*_e_,*T*_l_)-MDEF simulations to calculate, at each time step, the friction and stochastic forces in eq 4.

## 4 Results of the Dynamics Simulations

We have hitherto shown how our best developed EANN-PES (Section 2) and our atomwise additive electronic density function approach (Section 3) yield results in being close to the original 0.75 ML-CO/Pd(111) AIMDEF data set. Despite this being a reasonable quality check, it is not sufficient to prove that using both functions in MDEF calculations produces exactly the same results as in AIMDEF. This is due to two possible undesirable situations.^46^ First, MDEF trajectories may sample regions of the configurational space that, even when close to the EANN training set or the DGF data set, are not yet correctly described by the optimized parameters, and second, these trajectories may enter regions of the configurational space far from the AIMDEF data set where both fitted functions have to extrapolate information. In addition, the probability of encountering any of the described problems is increased by the high number of DOFs, in our case 108, that play a role during this type of laser-induced desorption dynamics simulations.

To rule out these sources of error and further address the accuracy of our developed EANN-PES and additive DGF, we have simulated the desorption of CO from 0.75 ML-CO/Pd(111) with (*T*_e_,*T*_l_)-MDEF, assuming the same experimental conditions as in the original (*T*_e_,*T*_l_)-AIMDEF calculations.^27^ Specifically, the Pd(111) being initially at 90 K is heated by a laser pulse of 780 nm wavelength, 100 fs of full width at half-maximum (FWHM), and absorbed fluence *F* = 13 mJ/cm^2^. In practice, the heated surface is modeled with the same time-dependent temperatures *T*_e_(*t*) and *T*_l_(*t*) used in the (*T*_e_,*T*_l_)-AIMDEF simulations,^27^ in which the maximum of the laser pulse arrives at the instant *t* = 410 fs.

Using these conditions, two sets of dynamics calculations have been run. In the first one, hereinafter called (*T*_e_,*T*_l_)-MDEF-1, we have used the same set of 100 initial configurations as utilized in ref (27). This allows us to compare step by step the extent to which (*T*_e_,*T*_l_)-MDEF and (*T*_e_,*T*_l_)-AIMDEF trajectories are similar and detect if there are artifacts in the potential or electronic friction coefficients that could push trajectories away from the EANN-PES confidence zone, even when starting from (*T*_e_,*T*_l_)-AIMDEF initial configurations. In the second set of calculations, hereinafter called (*T*_e_,*T*_l_)-MDEF-2, we have used 2000 configurations selected randomly from a set of 10 000 structures generated by letting the 100 initial configurations of ref (27) evolve in 1 ps with a constant temperature of 90 K. This enables us to test how robust (*T*_e_,*T*_l_)-MDEF dynamics is when trajectories start from configurations not included in any of the data sets used to fit the EANN-PES or DGF.

**Figure 8:**
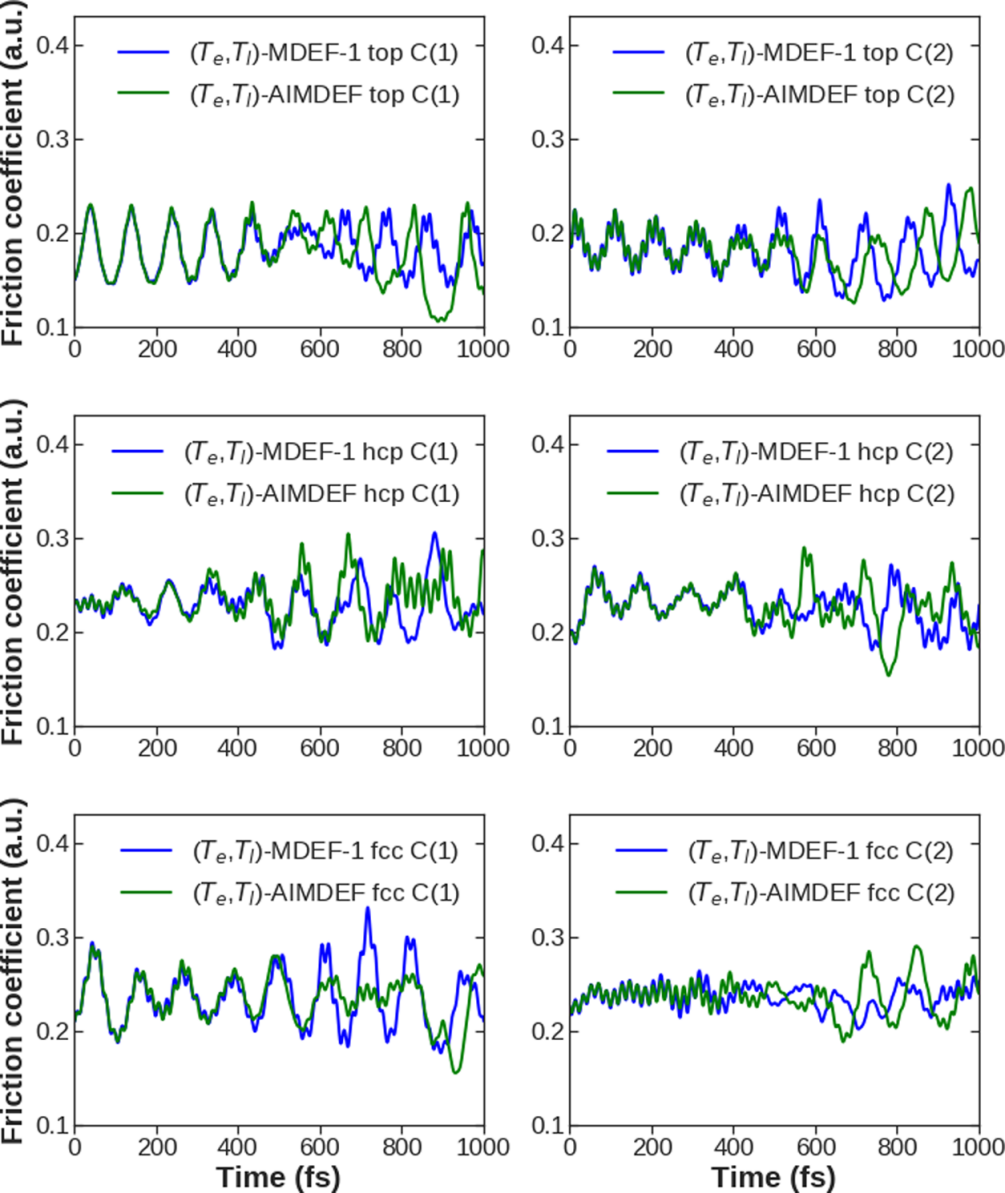
Friction coefficients of C atoms as a function of time for a typical trajectory. Green lines, (*T*_e_,*T*_l_)-AIMDEF results for a given trajectory extracted from ref (27). Blue lines, EANN results for a trajectory initiated with the same (*T*_e_,*T*_l_)-AIMDEF conditions. Each panel stands for a different C atom in the model. Top, fcc, and hcp sites refer to the C atoms’ initial positions.

We find that (*T*_e_,*T*_l_)-MDEF-1 trajectories lie close to their (*T*_e_,*T*_l_)-AIMDEF counterparts. In fact, configurations visited during the first 400–500 fs are practically identical to those visited by the original trajectories. To illustrate this finding, in Figure 8 we show how a typical (*T*_e_,*T*_l_)-MDEF-1 trajectory (blue line) compares with its corresponding (*T*_e_,*T*_l_)-AIMDEF trajectory (green line) in terms of time evolution of C atom friction coefficients during 1 ps. The agreement for times below 500 fs is very high, independent of the initial position of the C atoms. After 500 fs, friction coefficients start to diverge as trajectories commence to follow different pathways due to cumulative differences in the dynamics. This behavior is shared among all (*T*_e_,*T*_l_)-MDEF-1 simulations and reflects that the quality of our fitted EANN-PES and DGF is good enough to keep (*T*_e_,*T*_l_)-MDEF trajectories close to their (*T*_e_,*T*_l_)-AIMDEF analogues in a very detailed way for several hundred femtoseconds. This is a remarkable quality achievement as the agreement was attained despite the possible dynamical instability, the stochastic nature of the Langevin equations of motions, and the different integration time steps used in the simulations (Δ*t* = 0.2 fs in MDEF, Δ*t* = 1 fs in AIMDEF) for an amount of time in which the electronic temperature of the 2TM changes from 90 K (*t* = 0 fs) to 5500 K (*t* = 500 fs).

**Figure 9:**
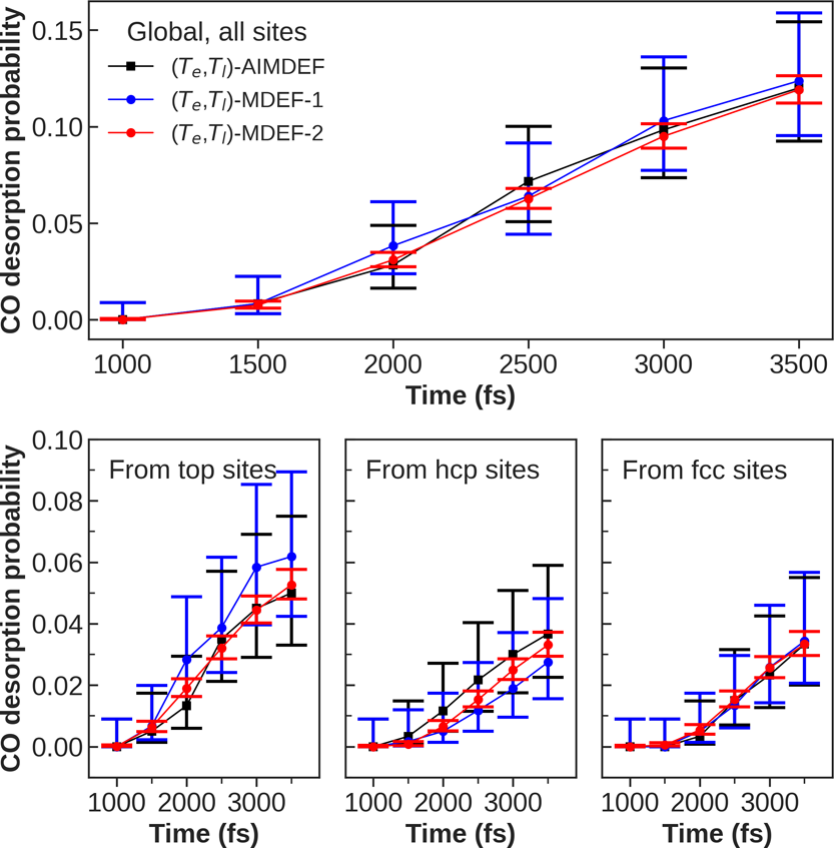
Top: global CO desorption probability as a function of time. Bottom: CO desorption probabilities ordered by the initial site as a function of time. For all panels, (*T*_e_,*T*_l_)-AIMDEF represents results extracted from ref (27) calculations (black), (*T*_e_,*T*_l_)-MDEF-1 represents dynamics results obtained from our best trained potential using the same 100 (*T*_e_,*T*_l_)-AIMDEF initial conditions (blue), and (*T*_e_,*T*_l_)-MDEF-2 represents dynamics results obtained with the same EANN potential using 2000 random initial conditions (red).

In Figure 9, we show (*T*_e_,*T*_l_)-MDEF-1 (blue line), (*T*_e_,*T*_l_)-MDEF-2 (red line), and (*T*_e_,*T*_l_)-AIMDEF (black line) results for total (top panel) and initial site-dependent (bottom panels) CO desorption probabilities as a function of time. In each case, the desorption probability *p*_CO_ is defined as the (cumulative) number of CO molecules desorbed at a given time divided by the total number of trajectories (*N*_traj_) and the total number of CO molecules in our simulation cell (six; see Figure 6). Since this definition is equivalent to consider that the number of CO desorption events distribute as a binomial distribution with six *N*_traj_ independent trials and success probability *p*_CO_, we associate to each evaluation of *p*_CO_ a confidence interval calculated with Wilson asymmetric score intervals^64^ for the confidence of 99%. It is apparent that all desorption probabilities calculated with (*T*_e_,*T*_l_)-MDEF-1 and (*T*_e_,*T*_l_)-MDEF-2 are in good agreement with the (*T*_e_,*T*_l_)-AIMDEF results, especially in the case of total and fcc-CO desorption probabilities. On a closer look, we can see that site-dependent (*T*_e_,*T*_l_)-MDEF-1 desorption probabilities present more discrepancies with (*T*_e_,*T*_l_)-AIMDEF than (*T*_e_,*T*_l_)-MDEF-2. In particular, (*T*_e_,*T*_l_)-MDEF-1 top-CO desorption ratios tend to be higher than those of (*T*_e_,*T*_l_)-AIMDEF, whereas hcp-CO desorption ratios tend to be lower. These deviations are mostly from the statistical variability associated with their respective 100 trajectories’ ensembles, since all (*T*_e_,*T*_l_)-MDEF-1 and (*T*_e_,*T*_l_)-AIMDEF desorption probabilities are within their 99% confidence intervals reciprocally. In the case of (*T*_e_,*T*_l_)-MDEF-2, all desorption probabilities lie closer to those of (*T*_e_,*T*_l_)-AIMDEF despite not sharing exactly the same initial configurations. This supports that our EANN-PES and DGF are accurate enough to describe the dynamic energy barriers that CO molecules encounter during the laser-induced desorption process on the same footing as the original AIMDEF calculations.

Compared to (*T*_e_,*T*_l_)-AIMDEF, the improved statistics of the (*T*_e_,*T*_l_)-MDEF-2 simulations allow us to establish with more precision the time scale of the desorption process. It takes more than 0.6 ps since the arrival of the laser-pulse maximum (occurring at *t* = 410 fs) to observe the first desorption events. The site-resolved desorption probabilities show that these initial events correspond to atop-CO adsorbates. Around an additional 500 fs are required for desorption from either the hcp or fcc sites.

**Figure 10:**
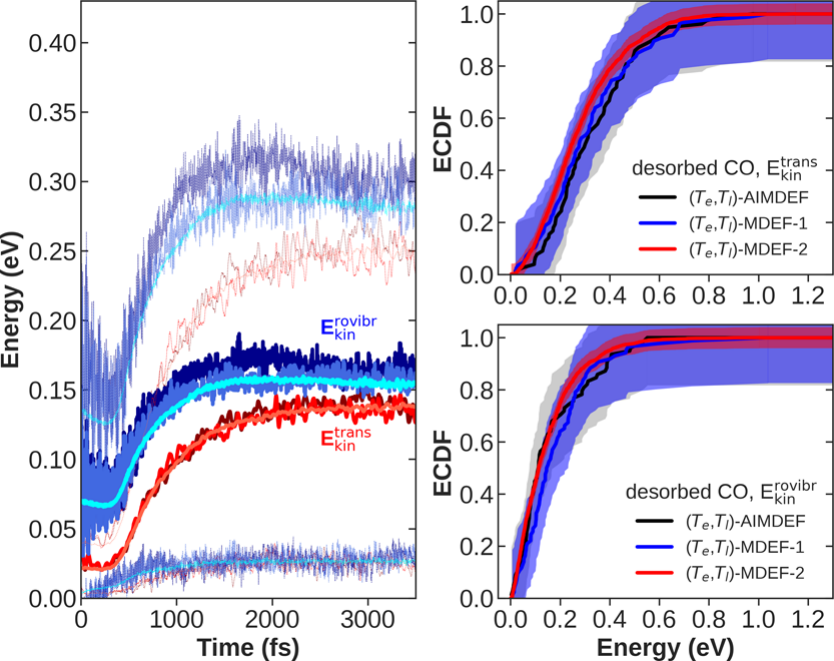
Left: mean center of mass translational kinetic energy, *E*_kin_^trans^ (reddish, full thick lines), and mean rovibrational kinetic energy, *E*_kin_^rovibr^ (bluish, full thick lines), of adsorbed CO molecules as a function of time. The corresponding thin dotted curves above and below each *E*_kin_^trans(rovibr)^ curve show the mean values plus and minus associated standard deviations, respectively. Right: center of mass translational energy (top) and rovibrational kinetic energy (bottom) empirical cumulative distribution functions (ECDFs) of desorbed CO molecules. Shaded areas mark 99% Dvoretzky–Kiefer–Wolfowitz confidence intervals.^65^ For all panels, (*T*_e_,*T*_l_)-AIMDEF represents results extracted from ref (27) calculations, (*T*_e_,*T*_l_)-MDEF-1 represents dynamics results obtained from our best trained potential using the same 100 (*T*_e_,*T*_l_)-AIMDEF initial conditions, and (*T*_e_,*T*_l_)-MDEF-2 represents dynamics results obtained with the same EANN potential using 2000 random initial conditions.

Having demonstrated the accuracy of our methodology to reproduce the (*T*_e_,*T*_l_)-AIMDEF trajectories for the first few hundred femtoseconds and the final outcome in terms of CO desorption probabilities, we now focus on the time evolution of the kinetic energy of CO molecules, which is more sensitive to the fine details of the paths followed on the PES. In Figure 10 (left panel), we plot the mean translational (*E*_kin_^trans^, reddish, full thick lines) and mean rovibrational (*E*_kin_^rovibr^, bluish, full thick lines) kinetic energy of adsorbed CO molecules averaged over trajectories as a function of time. We define *E*_kin_^trans^ as only the mean translational energy of the center of mass of CO molecules, while *E*_kin_^rovibr^ is calculated as the mean of C and O total kinetic energy minus *E*_kin_^trans^. Starting with *E*_kin_^trans^, we observe that the (*T*_e_,*T*_l_)-MDEF-1 (red) and (*T*_e_,*T*_l_)-MDEF-2 (light red) results lie very close to the (*T*_e_,*T*_l_)-AIMDEF (dark red) values, showing that the mean kinetic translational energies are very well reproduced by our EANN-PES. A similar conclusion is extracted when comparing the results for the mean rovibrational kinetic energies, since both the (*T*_e_,*T*_l_)-MDEF-1 (blue) and (*T*_e_,*T*_l_)-MDEF-2 (light blue) results agree remarkably well with the (*T*_e_,*T*_l_)-AIMDEF *E*_kin_^rovibr^ values (dark blue). But together with the mean energy values, it is important to confirm whether the instantaneous energy distributions of the adsorbates are also well reproduced by our EANN-PES. The latter can be estimated by comparing, for instance, the associated standard deviations of the translational Δ*E*_kin_^trans^ and rovibrational Δ*E*_kin_^trans^ kinetic energy distributions. Thus, there are two additional (thin dotted) curves representing the values *E*_kin_^trans(rovibr)^ ± Δ*E*_kin_^trans(rovibr)^ associated to each mean kinetic energy curve in Figure 10. As shown in the figure, there is also a good agreement between the (*T*_e_,*T*_l_)-AIMDEF instantaneous standard deviations and those obtained from the (*T*_e_,*T*_l_)-MDEF-1 and (*T*_e_,*T*_l_)-MDEF-2 simulations, which reinforces the high quality of the EANN-PES.

In Figure 10 (right panels), we also show the empirical cumulative distribution function (ECDF) of ensemble-averaged mean translational (top-right panel) and mean rovibrational (bottom-right panel) kinetic energies of desorbed CO molecules. These energy distributions are defined in the same way as in the previous panel, but they are only calculated when desorbed CO molecules are far away from the surface (center-of-mass-to-surface-plane distance greater than 6 Å) at the end of the dynamics (3500 fs). We have marked with shaded areas around each ECDF their associated 99% Dvoretzky–Kiefer–Wolfowitz confidence intervals.^65^ These areas define for each empirical distribution the region inside which their exact cumulative distribution functions lie with a 99% confidence. From these panels, we can see that (*T*_e_,*T*_l_)-MDEF-1 (blue line) and (*T*_e_,*T*_l_)-MDEF-2 (red line) evaluated mean energies agree considerably with (*T*_e_,*T*_l_)-AIMDEF (black) results, as all MDEF curves lie within the confidence interval of the original calculation. It is also apparent that the (*T*_e_,*T*_l_)-MDEF-2 confidence intervals are much smaller than the others (and therefore closer to the exact distribution), due to the higher number of trajectories sampled. These results further show that both, rovibration and translation of CO molecules on top of the surface and after desorption, are in good consonance with the findings in ref (27).

Taking advantage of the improved statistics provided by (*T*_e_,*T*_l_)-MDEF-2, we can reliably determine the rovibrational state of the desorbed CO molecules (ν_f_, *j*_f_). In performing such an analysis, the rotational quantum number *j*_f_ is computed from the classical angular momentum *L*_cl_ as the closest integer that verifies , while the vibrational state ν_f_ is determined from the vibrational action α_f_ as the nearest integer that verifies ν_f_ = α_f_/*h* – 1/2, where *h* is the Planck constant.^66^ The results from this quasi-classical analysis show that most of the desorbed molecules (83.3%) are in the vibrational ground state and slightly rotationally excited (*j*_f_ ≤ 20).

## 5 Conclusions

Using the recently developed embedded atomic neural network (EANN)^47^ framework, we generate an accurate and complex potential energy surface that is able to describe the dynamics of the femtosecond laser-induced desorption of CO from Pd(111) with a coverage of 0.75 ML. As the training data set, we use around 16 000 configurations taken from ab initio molecular dynamics simulations [(*T*_e_,*T*_l_)-AIMDEF]^27^ that incorporate both the effect of the laser-excited electrons and the concomitant excitation of the surface phonons. The otherwise random selection of AIMDEF configurations has been biased so that the final training set incorporates a defined proportion of configurations from trajectories characterized by a different number of CO desorption events (in practice, none, one, and two in the proportion 3:4:3, respectively). This procedure enforces the training set to contain an equilibrated amount of information about different desorption channels and different instantaneous surface CO coverages. The quality of our EANN-PES in predicting not only the system energy but also the atomic forces is validated against a huge set of almost 90 000 AIMDEF configurations not included in the training process. An impressively small RMSE of 0.85 meV is achieved in the energy per moving atom and of around 0.05 eV/Å for the forces.

The EANN-PES robustness is further checked by evaluating its performance in simulating the desorption dynamics of CO from the Pd(111) surface covered with 0.75 ML. Following the (*T*_e_,*T*_l_)-AIMDEF method, the excitation created by the laser in the electronic system and the subsequent excitation of the surface phonons is represented within the two-temperature model as two coupled thermal baths with temperatures *T*_e_(*t*) and *T*_l_(*t*). Next, the coupling of each adsorbate to the highly excited electrons is described by a Langevin equation that depends on the time-dependent electronic temperature *T*_e_(*t*), and the Nosé–Hoover thermostat is used to describe the time-dependent temperature of the surface atoms *T*_l_(*t*). In all these equations, the adiabatic forces over each adsorbate and surface atom, which are obtained from the Hellmann–Feynman theorem in AIMDEF, are here calculated by means of the 0.75 ML-CO/Pd(111) EANN-PES. The value of the density at the position of the adsorbates at each time step, necessary to evaluate the friction coefficients entering the nonadiabatic forces of the Langevin equation within the LDFA scheme, is obtained with an efficient DGF based on the fitting of the (*T*_e_,*T*_l_)-AIMDEF friction coefficients. In this way, the (*T*_e_,*T*_l_)-AIMDEF results are reproduced with a remarkable level of accuracy. This demonstrates the outstanding performance of the obtained EANN-PES that can cover an extensive range of surface temperatures (90–1000 K); a large number of degrees of freedom, those corresponding to multiple adsorbates and surface atoms, i.e., 108 in our simulation cell; and an extremely complex configurational space, characterized by very mobile adsorbates and a changing CO coverage caused by the sizable desorption.

Application of this NN-PES for future computational tests of system dynamics under different initial conditions should be straightforward. Up to now, molecular dynamics simulations at the ab initio-DFT level of femtosecond laser pulse-induced reactions at surfaces have been limited either by the impossibility to account for all the relevant degrees of freedom of the system or, in the case of (*T*_e_,*T*_l_)-AIMDEF calculations, by the huge computational cost of AIMD that restricted the achievable statistics. In this respect, the usefulness of NN-PES in the present work cannot be underestimated. For instance, it will allow us to perform simulations, at the level of (*T*_e_,*T*_l_)-AIMDEF with low computational costs and higher statistics, for different laser fluences and adsorbate coverages. Also, the theoretical study of the time delay dependence of two pulse correlation experiments will be tractable. It will be also possible to extend the simulation times to values much larger than the usual 2–4 ps in (*T*_e_,*T*_l_)-AIMDEF to guarantee that the computed reaction/desorption probabilities are saturated. The increase of the simulation cell to avoid finite-size effects and to account more accurately for interadsorbate energy exchange will also be easily accessible. Indeed, it opens the path to, from a theoretical point of view, fully characterize and understand these kinds of experiments. Last but not least, it must be stressed that our work constitutes a strong support for utilization of the EANN methodological framework for the development of accurate NN-PESs for other complex gas–solid interfaces.

## Notes

The PES generated during the current study is not publicly available due to ongoing projects but is available from the authors on reasonable request. B.J.’s group is preparing a Python version of the EANN code that will be accessible in the near future.

## References

[ref1] Frischkorn C.; Wolf M. Femtochemistry at Metal Surfaces: Nonadiabatic Reaction Dynamics. Chem. Rev. 2006, 106, 4207–4233. 10.1021/cr050161r. 17031984

[ref2] Guo H.; Saalfrank P.; Seideman T. Theory of photoinduced surface reactions of admolecules. Prog. Surf. Sci. 1999, 62, 239–303. 10.1016/S0079-6816(99)00013-1.

[ref3] Saalfrank P. Quantum Dynamical Approach to Ultrafast Molecular Desorption from Surfaces. Chem. Rev. 2006, 106, 4116–4159. 10.1021/cr0501691. 17031982

[ref4] Budde F.; Heinz T. F.; Loy M. M. T.; Misewich J. A.; de Rougemont F.; Zacharias H. Femtosecond time-resolved measurement of desorption. Phys. Rev. Lett. 1991, 66, 3024–3027. 10.1103/PhysRevLett.66.3024. 10043679

[ref5] Misewich J. A.; Heinz T. F.; Newns D. M. Desorption induced by multiple electronic transitions. Phys. Rev. Lett. 1992, 68, 3737–3740. 10.1103/PhysRevLett.68.3737. 10045784

[ref6] Bonn M.; Funk S.; Hess C.; Denzler D. N.; Stampfl C.; Scheffler M.; Wolf M.; Ertl G. Phonon- Versus Electron-Mediated Desorption and Oxidation of CO on Ru(0001). Science 1999, 285, 1042–1045. 10.1126/science.285.5430.1042. 10446045

[ref7] Denzler D. N.; Frischkorn C.; Hess C.; Wolf M.; Ertl G. Electronic Excitation and Dynamic Promotion of a Surface Reaction. Phys. Rev. Lett. 2003, 91, 226102 10.1103/PhysRevLett.91.226102. 14683251

[ref8] Szymanski P.; Harris A. L.; Camillone N. Adsorption-state-dependent subpicosecond photoinduced desorption dynamics. J. Chem. Phys. 2007, 126, 214709 10.1063/1.2735594. 17567215

[ref9] Hong S.-Y.; Xu P.; Camillone N. R.; White M. G.; Camillone N. III Adlayer structure dependent ultrafast desorption dynamics in carbon monoxide adsorbed on Pd (111). J. Chem. Phys. 2016, 145, 014704 10.1063/1.4954408. 27394118

[ref10] Vazhappilly T.; Klamroth T.; Saalfrank P.; Hernandez R. Femtosecond-Laser Desorption of H2 (D2) from Ru(0001): Quantum and Classical Approaches. J. Phys. Chem. C 2009, 113, 7790–7801. 10.1021/jp810709k.

[ref11] Füchsel G.; Klamroth T.; Tremblay J. C.; Saalfrank P. Stochastic Approach to Laser-Induced Ultrafast Dynamics: The Desorption of H2/D2 from Ru(0001). Phys. Chem. Chem. Phys. 2010, 12, 14082–14094. 10.1039/c0cp00895h. 20856974

[ref12] Füchsel G.; Klamroth T.; Monturet S.; Saalfrank P. Dissipative Dynamics within the Electronic Friction Approach: The Femtosecond Laser Desorption of H2/D2 from Ru(0001). Phys. Chem. Chem. Phys. 2011, 13, 8659–8670. 10.1039/c0cp02086a. 21369575

[ref13] Lončarić I.; Alducin M.; Saalfrank P.; Juaristi J. I. Femtosecond-Laser-Driven Molecular Dynamics on Surfaces: Photodesorption of Molecular Oxygen from Ag(110). Phys. Rev. B 2016, 93, 014301 10.1103/PhysRevB.93.014301.

[ref14] Lončarić I.; Alducin M.; Saalfrank P.; Juaristi J. I. Femtosecond Laser Pulse Induced Desorption: a Molecular Dynamics Simulation. Nucl. Instrum. Methods Phys. Res., Sect. B 2016, 382, 114–118. 10.1016/j.nimb.2016.02.051.

[ref15] Scholz R.; Floß G.; Saalfrank P.; Füchsel G.; Lončarić I.; Juaristi J. I. Femtosecond-Laser Induced Dynamics of CO On Ru(0001): Deep Insights from a Hot-Electron Friction Model Including Surface Motion. Phys. Rev. B 2016, 94, 165447 10.1103/PhysRevB.94.165447.

[ref16] Lončarić I.; Füchsel G.; Juaristi J. I.; Saalfrank P. Strong Anisotropic Interaction Controls Unusual Sticking and Scattering of CO at Ru(0001). Phys. Rev. Lett. 2017, 119, 146101 10.1103/PhysRevLett.119.146101. 29053313

[ref17] Scholz R.; Lindner S.; Lončarić I.; Tremblay J. C.; Juaristi J. I.; Alducin M.; Saalfrank P. Vibrational response and motion of carbon monoxide on Cu(100) driven by femtosecond laser pulses: Molecular dynamics with electronic friction. Phys. Rev. B 2019, 100, 245431 10.1103/PhysRevB.100.245431.

[ref18] Anisimov S. I.; Kapeliovich B. L.; Perel’man T. L. Electron emission from metal surfaces exposed to ultrashort laser pulses. Sov. Phys.-JETP 1974, 39, 375.

[ref19] Alducin M.; Díez Muiño R.; Juaristi J. I. Non-adiabatic effects in elementary reaction processes at metal surfaces. Prog. Surf. Sci. 2017, 92, 317–340. 10.1016/j.progsurf.2017.09.002.

[ref20] Blanco-Rey M.; Juaristi J. I.; Díez Muiño R.; Busnengo H. F.; Kroes G. J.; Alducin M. Electronic Friction Dominates Hydrogen Hot-Atom Relaxation on Pd(100). Phys. Rev. Lett. 2014, 112, 103203 10.1103/PhysRevLett.112.103203. 24679290

[ref21] Saalfrank P.; Juaristi J. I.; Alducin M.; Blanco-Rey M.; Díez Muiño R. Vibrational Lifetimes of Hydrogen on Lead Films: An Ab Initio Molecular Dynamics with Electronic Friction (AIMDEF) Study. J. Chem. Phys. 2014, 141, 234702 10.1063/1.4903309. 25527952

[ref22] Novko D.; Blanco-Rey M.; Juaristi J. I.; Alducin M. *Ab Initio* Molecular Dynamics with Simultaneous Electron and Phonon Excitations: Application To The Relaxation of Hot Atoms and Molecules On Metal Surfaces. Phys. Rev. B 2015, 92, 201411 10.1103/PhysRevB.92.201411.

[ref23] Novko D.; Blanco-Rey M.; Alducin M.; Juaristi J. I. Surface electron density models for accurate ab initio molecular dynamics with electronic friction. Phys. Rev. B 2016, 93, 245435 10.1103/PhysRevB.93.245435.

[ref24] Novko D.; Blanco-Rey M.; Juaristi J. I.; Alducin M. Energy Loss in Gas-Surface Dynamics: Electron-Hole Pair and Phonon Excitation Upon Adsorbate Relaxation. Nucl. Instrum. Methods Phys. Res., Sect. B 2016, 382, 26–31. 10.1016/j.nimb.2016.02.031.

[ref25] Novko D.; Lončarić I.; Blanco-Rey M.; Juaristi J. I.; Alducin M. Energy loss and surface temperature effects in ab initio molecular dynamics simulations: N adsorption on Ag(111) as a case study. Phys. Rev. B 2017, 96, 085437 10.1103/PhysRevB.96.085437.

[ref26] Juaristi J. I.; Alducin M.; Saalfrank P. Femtosecond laser induced desorption of H_2_,D_2_, and HD from Ru(0001): Dynamical promotion and suppression studied with ab initio molecular dynamics with electronic friction. Phys. Rev. B 2017, 95, 125439 10.1103/PhysRevB.95.125439.

[ref27] Alducin M.; Camillone N.; Hong S.-Y.; Juaristi J. I. Electrons and Phonons Cooperate in the Laser-Induced Desorption of CO from Pd(111). Phys. Rev. Lett. 2019, 123, 246802 10.1103/PhysRevLett.123.246802. 31922860

[ref28] Jiang B.; Li J.; Guo H. High-Fidelity Potential Energy Surfaces for Gas-Phase and Gas-Surface Scattering Processes from Machine Learning. J. Phys. Chem. Lett. 2020, 11, 5120–5131. 10.1021/acs.jpclett.0c00989. 32517472

[ref29] Jiang B.; Guo H. Dynamics of Water Dissociative Chemisorption on Ni(111): Effects of Impact Sites and Incident Angles. Phys. Rev. Lett. 2015, 114, 166101 10.1103/PhysRevLett.114.166101. 25955057

[ref30] Shen X.; Chen J.; Zhang Z.; Shao K.; Zhang D. H. Methane dissociation on Ni(111): A fifteen-dimensional potential energy surface using neural network method. J. Chem. Phys 2015, 143, 144701 10.1063/1.4932226. 26472389

[ref31] Kolb B.; Luo X.; Zhou X.; Jiang B.; Guo H. High-Dimensional Atomistic Neural Network Potentials for Molecule Surface Interactions: HCl Scattering from Au(111). J. Phys. Chem. Lett. 2017, 8, 666–672. 10.1021/acs.jpclett.6b02994. 28102689

[ref32] Shakouri K.; Behler J.; Meyer J.; Kroes G.-J. Accurate Neural Network Description of Surface Phonons in Reactive Gas-Surface Dynamics: N2 + Ru(0001). J. Phys. Chem. Lett. 2017, 8, 2131–2136. 10.1021/acs.jpclett.7b00784. 28441867 PMC5439174

[ref33] Liu Q.; Zhou X.; Zhou L.; Zhang Y.; Luo X.; Guo H.; Jiang B. Constructing High-Dimensional Neural Network Potential Energy Surfaces for Gas-Surface Scattering and Reactions. J. Phys. Chem. C 2018, 122, 1761–1769. 10.1021/acs.jpcc.7b12064.

[ref34] Chen J.; Zhou X.; Zhang Y.; Jiang B. Vibrational control of selective bond cleavage in dissociative chemisorption of methanol on Cu(111). Nat. Commun. 2018, 9, 4039 10.1038/s41467-018-06478-6. 30279479 PMC6168487

[ref35] Shakouri K.; Behler J.; Meyer J.; Kroes G.-J. Analysis of Energy Dissipation Channels in a Benchmark System of Activated Dissociation: N2 on Ru(0001). J. Phys. Chem. C 2018, 122, 23470–23480. 10.1021/acs.jpcc.8b06729. PMC6196344 30364480

[ref36] Gerrits N.; Shakouri K.; Behler J.; Kroes G.-J. Accurate Probabilities for Highly Activated Reaction of Polyatomic Molecules on Surfaces Using a High-Dimensional Neural Network Potential: CHD3 + Cu(111). J. Phys. Chem. Lett. 2019, 10, 1763–1768. 10.1021/acs.jpclett.9b00560. 30922058 PMC6477808

[ref37] Zhang Y.; Zhou X.; Jiang B. Bridging the Gap between Direct Dynamics and Globally Accurate Reactive Potential Energy Surfaces Using Neural Networks. J. Phys. Chem. Lett. 2019, 10, 1185–1191. 10.1021/acs.jpclett.9b00085. 30802067

[ref38] Huang M.; Zhou X.; Zhang Y.; Zhou L.; Alducin M.; Jiang B.; Guo H. Adiabatic and nonadiabatic energy dissipation during scattering of vibrationally excited CO from Au(111). Phys. Rev. B 2019, 100, 201407 10.1103/PhysRevB.100.201407.

[ref39] Rivero Santamaría A.; Ramos M.; Alducin M.; Busnengo H. F.; Díez Muiño R.; Juaristi J. I. High-Dimensional Atomistic Neural Network Potential to Study the Alignment-Resolved O2 Scattering from Highly Oriented Pyrolytic Graphite. J. Phys. Chem. A 2021, 125, 2588–2600. 10.1021/acs.jpca.1c00835. 33734696

[ref40] Natarajan S. K.; Behler J. Neural network molecular dynamics simulations of solid-liquid interfaces: water at low-index copper surfaces. Phys. Chem. Chem. Phys. 2016, 18, 28704–28725. 10.1039/C6CP05711J. 27722603

[ref41] Quaranta V.; Hellström M.; Behler J. Proton-Transfer Mechanisms at the Water ZnO Interface: The Role of Presolvation. J. Phys. Chem. Lett. 2017, 8, 1476–1483. 10.1021/acs.jpclett.7b00358. 28296415

[ref42] Hellström M.; Quaranta V.; Behler J. One-dimensional vs. two-dimensional proton transport processes at solid-liquid zinc-oxide-water interfaces. Chem. Sci. 2019, 10, 1232–1243. 10.1039/C8SC03033B. 30774924 PMC6349017

[ref43] Ghorbanfekr H.; Behler J.; Peeters F. M. Insights into Water Permeation through hBN Nanocapillaries by Ab Initio Machine Learning Molecular Dynamics Simulations. J. Phys. Chem. Lett. 2020, 11, 7363–7370. 10.1021/acs.jpclett.0c01739. 32787306

[ref44] Behler J.; Parrinello M. Generalized neural-network representation of high-dimensional potential-energy surfaces. Phys. Rev. Lett. 2007, 98, 146401 10.1103/PhysRevLett.98.146401. 17501293

[ref45] Behler J. Representing potential energy surfaces by high-dimensional neural network potentials. J. Phys.: Condens. Matter 2014, 26, 183001 10.1088/0953-8984/26/18/183001. 24758952

[ref46] Behler J. Constructing High-Dimensional Neural Network Potentials: A Tutorial Review. Int. J. Quantum Chem. 2015, 115, 1032–1050. 10.1002/qua.24890.

[ref47] Zhang Y.; Hu C.; Jiang B. Embedded Atom Neural Network Potentials: Efficient and Accurate Machine Learning with a Physically Inspired Representation. J. Phys. Chem. Lett. 2019, 10, 4962–4967. 10.1021/acs.jpclett.9b02037. 31397157

[ref48] Zhu L.; Zhang Y.; Zhang L.; Zhou X.; Jiang B. Unified and transferable description of dynamics of H2 dissociative adsorption on multiple copper surfaces via machine learning. Phys. Chem. Chem. Phys. 2020, 22, 13958–13964. 10.1039/D0CP02291H. 32609134

[ref49] Zhou X.; Zhang Y.; Guo H.; Jiang B. Towards bridging the structure gap in heterogeneous catalysis: the impact of defects in dissociative chemisorption of methane on Ir surfaces. Phys. Chem. Chem. Phys. 2021, 23, 4376–4385. 10.1039/D0CP06535H. 33592080

[ref50] Gastegger M.; Schwiedrzik L.; Bittermann M.; Berzsenyi F.; Marquetand P. wACSF–Weighted atom-centered symmetry functions as descriptors in machine learning potentials. J. Chem. Phys. 2018, 148, 241709 10.1063/1.5019667. 29960372

[ref51] Kresse G.; Furthmüller J. Efficiency of Ab-Initio Total Energy Calculations For Metals and Semiconductors Using a Plane-Wave Basis Set. Comput. Mater. Sci. 1996, 6, 15–50. 10.1016/0927-0256(96)00008-0. 9984901

[ref52] Kresse G.; Furthmüller J. Efficient Iterative Schemes For Ab Initio Total-Energy Calculations Using a Plane-Wave Basis Set. Phys. Rev. B 1996, 54, 11169–11186. 10.1103/PhysRevB.54.11169. 9984901

[ref53] Dion M.; Rydberg H.; Schröder E.; Langreth D. C.; Lundqvist B. I. Van der Waals Density Functional for General Geometries. Phys. Rev. Lett. 2004, 92, 246401 10.1103/PhysRevLett.92.246401. 15245113

[ref54] Zhang Y.; Zhou X.; Jiang B. Accelerating the Construction of Neural Network Potential Energy Surfaces: A Fast Hybrid Training Algorithm. Chin. J. Chem. Phys. 2017, 30, 727–734. 10.1063/1674-0068/30/cjcp1711212.

[ref55] Nosé S. A unified formulation of the constant temperature molecular dynamics methods. J. Chem. Phys. 1984, 81, 511–519. 10.1063/1.447334.

[ref56] Hoover W. G. Canonical dynamics: Equilibrium phase-space distributions. Phys. Rev. A 1985, 31, 1695–1697. 10.1103/PhysRevA.31.1695. 9895674

[ref57] Hünenberger P. H. In Advanced Computer Simulation: Approaches for Soft Matter Sciences I; Holm C.; Kremer K., Eds.; Springer: Berlin, Heidelberg, 2005; pp 105–149.

[ref58] Juaristi J. I.; Alducin M.; Díez Muiño R.; Busnengo H. F.; Salin A. Role of Electron-Hole Pair Excitations in the Dissociative Adsorption of Diatomic Molecules on Metal Surfaces. Phys. Rev. Lett. 2008, 100, 116102 10.1103/PhysRevLett.100.116102. 18517799

[ref59] Hirshfeld F. L. Bonded-Atom Fragments For Describing Molecular Charge Densities. Theor. Chim. Acta 1977, 44, 129 10.1007/BF00549096.

[ref60] Yin R.; Zhang Y.; Jiang B. Strong Vibrational Relaxation of NO Scattered from Au(111): Importance of the Adiabatic Potential Energy Surface. J. Phys. Chem. Lett. 2019, 10, 5969–5974. 10.1021/acs.jpclett.9b01806. 31538787

[ref61] Spiering P.; Meyer J. Testing Electronic Friction Models: Vibrational De-excitation in Scattering of H2 and D2 from Cu(111). J. Phys. Chem. Lett. 2018, 9, 1803–1808. 10.1021/acs.jpclett.7b03182. 29528648 PMC5890313

[ref62] Spiering P.; Shakouri K.; Behler J.; Kroes G.-J.; Meyer J. Orbital-Dependent Electronic Friction Significantly Affects the Description of Reactive Scattering of N2 from Ru(0001). J. Phys. Chem. Lett. 2019, 10, 2957–2962. 10.1021/acs.jpclett.9b00523. 31088059 PMC6558642

[ref63] Zhang Y.; Maurer R. J.; Jiang B. Symmetry-Adapted High Dimensional Neural Network Representation of Electronic Friction Tensor of Adsorbates on Metals. J. Phys. Chem. C 2020, 124, 186–195. 10.1021/acs.jpcc.9b09965.

[ref64] Wilson E. B. Probable Inference, the Law of Succession, and Statistical Inference. J. Am. Stat. Assoc. 1927, 22, 209–212. 10.1080/01621459.1927.10502953.

[ref65] Massart P. The tight constant in the Dvoretzky-Kiefer-Wolfowitz inequality. Ann. Probab. 1990, 18, 1269–1283. 10.1214/aop/1176990746.

[ref66] Kroes G. J.; Pijper E.; Salin A. Dissociative chemisorption of H2 on the Cu(110) surface: A quantum and quasiclassical dynamical study. J. Chem. Phys. 2007, 127, 164722 10.1063/1.2798112. 17979386

